# Assessment of Health-Related Quality of Life Among Palestinian Adolescents With Type 1 Diabetes: A Cross-Sectional Investigation

**DOI:** 10.1155/pedi/3568243

**Published:** 2025-02-14

**Authors:** Farah Sadder, Maysaa Nemer

**Affiliations:** Institute of Community and Public Health, Birzeit University, West Bank, Birzeit P.O. Box 14, State of Palestine

**Keywords:** adolescents, health-related quality of life, Palestine, type 1 diabetes

## Abstract

**Objectives:** Type 1 diabetes (T1D) with its worldwide increasing incidence is one of the most serious chronic conditions of adolescence. This study aimed to assess the Palestinian adolescent diabetic patients' health-related quality of life (HRQOL) and to identify specific factors that could predict poor quality of life. We also aimed to compare adolescents' reported HRQOL to proxy reports by their parents.

**Methods:** A cross-sectional study was carried out between November 2022 and October 2023 in the six governorates of northern West Bank/Palestine: Jenin, Nablus, Qalqilya, Salfit, Tubas, and Tulkarm. Patients who were diagnosed with T1D for over 6 months from their recruitment, aged between 10 and 18 years, were recruited from diabetes clinics of the Ministry of Health (MOH) and the Palestine Diabetes Institute (PDI). One hundred seventy adolescents and 170 parents (or guardians) completed the Pediatric Quality of Life Inventory (Peds QL) 3.2 Diabetes Module for adolescents and parents, respectively.

**Results:** An acceptable mean of 70.6 for the total score was reported for the Peds QL 3.2 Diabetes Module. Better scores were reported for the diabetes management summary score compared to the diabetes symptom summary score. Worry and communication were the lowest and highest reported subscores, respectively. Parents reported significantly lower results than adolescents. Income, gender, and hemoglobin A1c (HbA1c) were the main predictors of HRQOL among adolescents with T1D in Palestine.

**Conclusions:** Future national health strategies should consider income differences and try to overcome health gaps among adolescents with T1D coming from low-income families. Future research is needed to explore the political and cultural aspects and their effects on HRQOL among diabetic adolescents in Palestine.

## 1. Introduction

Type 1 diabetes (T1D) is one of the most common and serious chronic conditions affecting children and adolescents around the world [1]. Although global reports on the incidence and prevalence of T1D may vary between countries, it is agreed that both are substantial and growing [2]. Expectedly, the burden of this rising epidemic will largely affect low- and lower-middle-income countries [3]. In Palestine, although no accurate national numbers are reported on the prevalence and incidence of T1D, the disease is reported to be following the global trend [4].

T1D is a life-long condition that requires complex and demanding management to prevent or delay complications [5]. This includes multiple daily insulin injections, close monitoring of blood glucose levels, physical exercise, and dietary planning, among others [5, 6], All of which aim at maintaining good health, adequate metabolic control, and preventing long-term complications [5, 6].

In recent years, there has been a shift in medical practice, as focus has extended from considering these medical parameters as a sole indicator of health to a more holistic approach that also accounts for the psychosocial aspects of living with T1D [7, 8]. Consequently, more efforts have been made to evaluate health-related quality of life (HRQOL) among patients with T1D [8, 9].

Several definitions are available for HRQOL; in general, it is agreed that HRQOL describes how the effects of a disease and/or its treatment are perceived by patients [8]. Numerous benefits have been reported highlighting the importance of evaluating HRQOL among patients with T1D to the extent that it is recommended to evaluate HRQOL outcomes routinely in clinical practice [9, 10]. These benefits include achieving good diabetes care and promotion of healthy coping [10].

When using glycemic control as a reflection to evaluate diabetes management or care, adolescents with T1D appear to be the age group that is frequently reported to have the worst glycemic control [11, 12]. Moreover, as reported by the American Diabetes Association (ADA), only about 20% of adolescents with T1D achieve the target hemoglobin A1c (HbA1c) [11]. Adolescence, defined by the World Health Organization as the second decade of life (age 10–19 years), is a transit time from childhood to adulthood [13]. During this life period, the accompanying physical, hormonal, cognitive, and psychosocial changes are responsible in part for the lower glycemic control among adolescents with diabetes [11, 14, 15]. In addition, it is during this time that adolescents start achieving autonomy, independence, and self-identification away from their family [16]. During this developmental period of life, the psychosocial aspects greatly influence the decisions adolescents with diabetes make which ultimately affect their diabetes care and quality of life [6, 11, 16].

Assessment of HRQOL among adolescents with T1D has recently increased around the world [8, 10]. Several case–control studies found that HRQOL among T1D adolescents is lower than that of their peers who are not diabetics [17, 18]. Different validated tools were used to assess HRQOL among adolescents with T1D [19, 20]. The studies varied in the used tools: being intended for adolescents with chronic conditions [18], being specifically designed for diabetic adolescents [18, 20], or combining different tools together to evaluate HRQOL among adolescents with T1D [15, 18, 20]. Overall, it is evident that using tools that are specifically designed for adolescents with diabetes allows for better identification of factors unique to these patients influencing HRQOL [8, 18, 20]. The Pediatric Quality of Life Inventory (Peds QL) Diabetes Module is one of the known tools tailored for evaluating HRQOL among children and young adults with T1D [18].

Parents' perspectives on their children's HRQOL have an influence on their utilization of healthcare services, clinical decision-making, and home treatment regimens [21, 22]. Moreover, research shows that there is often a difference in reported HRQOL between adolescents and their parents [23–26]. Therefore, parents' perceptions on their adolescents' HRQOL are evaluated through proxy reports [9, 20].

According to recent studies from the Arab World, HRQOL was evaluated among adolescents with T1D in several countries including Jordan, Kuwait, Saudi Arabia, and Egypt [23, 24, 27–29]. Female adolescents had significantly lower HRQOL in Kuwait, Jordan, and Saudi Arabia compared to males [23, 24, 28]. Higher HbA1c levels were associated with lower HRQOL in the region [23, 28, 29]. Palestine, however, has only one published study on this topic, which compared HRQOL of adolescents with diabetes to a matching control group [30]. The study found no difference between cases and controls' results, and both reported acceptable HRQOL [30]. However, this study did not use the diabetes-specific module for evaluating quality of life among adolescents with diabetes and did not report parent perceptions [30]. Therefore, there was a need for a more comprehensive study that includes both adolescents and their parents, using a standardized validated tool to evaluate HRQOL among diabetic adolescent patients in Palestine.

This study aimed to assess the Palestinian adolescent diabetic patients' HRQOL, to identify specific socioeconomic, diabetes-related, health service-related, and other specific factors that could be associated with poor quality of life. Furthermore, we aimed to compare adolescents reported HRQOL to proxy reports by their parents.

## 2. Methods

### 2.1. Study Design and Setting

This cross-sectional study was carried out between November 2022 and October 2023 in the six governorates of northern West Bank/Palestine: Jenin, Nablus, Qalqilya, Salfit, Tubas, and Tulkarm.

### 2.2. Population and Sample

Adolescents who were diagnosed with T1D for over 6 months at the time of recruitment, aged between 10 and 18 years, living in northern governorates of West Bank Palestine, and did not have other chronic conditions of intellectual impairment were recruited from diabetes clinics of the Ministry of Health (MOH) and the Palestine Diabetes Institute (PDI).

Unfortunately, to date, there is no documentation on the national number for adolescents diagnosed with T1D in the West Bank. After contacting the main healthcare providers in Palestine (MOH, UNRWA, PDI), we were able to arrive at an estimated number of less than 1000 in the northern governorates. A sample of 190 patients and 190 parents (or guardians) was invited to participate through means of convenient and purposive sampling aiming to capture a representative sample in terms of sex, age, location, and duration of diabetes, among others.

### 2.3. Procedures

Ethical approval was obtained from the Ethics Review Committee at the Institute of Community and Public Health/Birzeit University (Ref. 2022 [7-1]). Accordingly, permissions from the healthcare provider facilities involved, MOH and PDI, were granted. Eligible diabetic adolescents and their parents were then invited to participate in the study either during the waiting time at the clinic visits or through phone calls. After explaining the study aims and procedures, informed verbal consent and assent were obtained from parents and adolescents. Thereafter, data were collected through in-clinic or over-the-phone interviews based on patients' convenience. Questionnaires were completed on electronic devices using the KoBo Collect Toolbox [31]. Interviews with participants were conducted by two trained fieldworkers and the first author. Each interview lasted for 20 min.

### 2.4. Tools

Data were collected through a structured questionnaire. The questionnaire was divided into two sections. The first section included questions about socioeconomic, demographic, and medical information regarding disease duration, type of insulin delivery, and frequency of self-monitoring of blood glucose (SMBG), among other questions. The second section included HRQOL questionnaire, in which the Peds QL 3.2 Diabetes Module was used [32]. The validated Arabic version of this tool was used with permission from the Mapi Research Institute [9, 33]. This tool includes 33 items inquiring about diabetes symptoms (15 items), treatment barriers (5 items), treatment adherence (6 items), worry (3 items), and communication (4 items) [32]. A 5-point scoring scale was used for each item (from 0 = never a problem to 4 = always a problem). The Peds QL 3.2 Diabetes Module provides two forms for our target age group: the child report for ages 10–12 years and the teen report for ages 13–18 years. However, the Arabic translations for both reports were exactly the same. After completing the structuring of the questionnaire, it was piloted to ensure patient understanding. A similar two-section questionnaire was used for collecting data from parents. The first section included demographic data about the interviewed parent and family. The second section included the translated Arabic version of the parent proxy report of the Peds QL 3.2 Diabetes Module. This proxy report had the same 33 items of the adolescents' report with language modifications based on first- or third-person tense.

### 2.5. Data Analysis

Univariate descriptive analysis was used to summarize demographic and clinical information of diabetic adolescents. Categorical variables were reported as count and percentages *n* (%), while numerical variables were reported as mean and standard deviation (SD). For HbA1c levels, based on the ADA recommended HbA1c goal of less than 7.5%, patients were categorized as having controlled (HbA1c < 7.5%) and uncontrolled diabetes (HbA1c ≥ 7.5%) [14]. Regarding frequency of SMBG, the three to four times per day minimum frequency for SMBG recommendation by the International Society for Pediatric and Adolescent Diabetes (ISPAD) for low-resource settings was adopted [16]. For the Peds QL score, items were reverse scored and linearly transformed to a 0–100 scale (0 = 100, 1 = 75, 2 = 50, 3 = 25, and 4 = 0), such that higher scores indicate better self-perceived health. The total score of a scale is the sum of all the items divided by the number of items answered on all the scales. Higher scores indicated better quality of life. The total score and two summary subscores were used for evaluating HRQOL among diabetic adolescents. The diabetes symptom summary score (first 15 items) and the diabetes management summary score (the remainder of the items) were calculated. When tested for normality using the Shapiro–Wilk test, both adolescents and parents scores were negatively skewed. To compare the Peds QL total and summary scores with the scores from parent reports, the nonparametric Wilcoxon–Mann–Whitney test was used. Bivariate analysis using Mann–Whitney *U* and Kruskal–Wallis tests was conducted to test for differences in the HRQOL among different groups of adolescents based on demographic and diabetes-related differences. Afterward, a generalized linear model (GLM) (gamma with log link) was done to identify potential predictors of adolescents' HRQOL. Variables that showed significant differences in the Peds QL scores in the bivariate analysis and other variables that showed a strong but not significant difference and were also reported in previous studies as significant predictors of HRQOL of diabetic adolescents were included in the GLM. A *p* value of ≤0.05 was considered statistically significant in all tests. Data were analyzed using the Statistical Package for the Social Sciences (SPSS) program for Windows version 27.

## 3. Results

### 3.1. Demographics

Out of the 190 patients who were invited, 170 patients agreed to participate in the study, giving us a response rate of 89.5%. Among all participating patients, 47.1% were interviewed in clinics, and 52.9% were interviewed over the phone. The demographic characteristics of the patients are presented in Table 1. A gender distribution of 100 (58.8%) females and 70 (41.2%) males was represented. The mean age of participants was 13.58 ± 2.3. Patient distribution based on living areas was 54 (31.8%), 103 (60.6%), and 13 (7.6%) for rural, urban, and camp areas, respectively. For each participant, one parent (or guardian) completed the parent proxy report. Among the parents, 152 (89.4%) were females, and 18 (10.6%) were males. The mean age of the parents was 42 ± 6.9. As for their relation to the adolescents, 151 (88.8%), 17 (10%), and 2 (1.2%) represented mothers, fathers, and a sibling, respectively. Only 37.6% of the parents had university education, and the majority (72.9%) did not work. As for the monthly family income, 18.2% reported having an income of less than 2000 NIS per month, 67.1% and 14.7% reported having a monthly income between 2000 and 5000 NIS, and more than 5000 NIS, respectively.

### 3.2. Diabetes and Its Control

The mean duration of diabetes was 4.7 ± 3.2 years. The average of the last reported HbA1c was 9.3% ± 2.2%, and 79.9% of the patients in this study were considered uncontrolled. Most of the participants (86.5%) were taking insulin by prefilled pens, while 6.5% had insulin pumps, and 7.1% were using conventional syringes. Only 17.1% of the patients reported having either diabetes complications like kidney problems or hyperlipidemia or other chronic conditions like celiac disease or asthma. Moreover, 11.2% reported taking medications other than insulin. The majority of the adolescents, 148 (87.1%), had medical insurance, seven patients were not insured (4.1%), and 15 (8.8%) had multiple health insurances. As for the diabetes medication provider, 31 (18.2%) patients got their insulin from either governmental or UN dispensing pharmacies, 29 (17.1%) got it from private pharmacies, only 3 (1.8%) patients depended only on donations as a source of insulin, and the majority 107 (62.9%) depended on more than one provider for their insulin. Regarding frequency of SMBG, 61 (35.9%) of adolescents reported testing less than the recommended three to four times per day, while 109 (64.1%) checked their glucose level three to four times or more per day. Moreover, only 21 (12.4%) of the adolescents reported having a sensor for continuous blood glucose monitoring in the past month.

### 3.3. Adolescents' HRQOL

Described in Table 2 are the results of the HRQOL for diabetic adolescents as reported by patient and parent tools. The mean total score as reported by adolescents was 70.61. The mean results for the diabetes symptoms summary score and the diabetes management summary score were 64.37 and 75.90, respectively. Worry subscore was the lowest reported by adolescents 62.01, while communication subscore was the highest at 82.94. Parents reported significantly lower scores than adolescent patients in the diabetes management summary score 71.36 (*p*  < 0.001) and total score 68.21 (*p*  < 0.009) but not in the diabetes symptom summary score 64.5 (*p*=0.93). Treatment barriers and treatment adherence were the lowest and highest subscores reported by parents 61.73 and 78.08, respectively.

### 3.4. Factors Associated With HRQOL Among Adolescents With T1D

When compared with the adolescents' Peds QL scores, gender and income were the only variables that were significantly associated in the bivariate analysis. Females scored lower than males in all scores. However, significant differences were only noted in the diabetes management summary score (*p*=0.025), worry (*p*=0.030), and treatment adherence (*p*=0.039) subscores. Higher family monthly income was associated with better HRQOL in all scores, though it was only significant in the diabetes management summary score (*p*=0.031), total score (*p*=0.010), worry (*p*=0.002), and treatment adherence (*p*=0.049) subscores. There were no significant differences between adolescents who lived in urban, rural, or camp areas. Insulin delivery method was not found to be associated with reported HRQOL. As for the glycemic control, adolescents with controlled HbA1c reported better than those who were uncontrolled in both summary scores and total score. However, the difference was not found to be significant in the bivariate analysis. When included in the multivariate GLM along with gender, income, age, duration of diabetes, and frequency of glucose monitoring, HbA1c (as a continuous variable) was found to be a significant predictor of diabetes symptom summary score and the total score (Table 3). Gender differences were also present in the GLM, where male gender was a significant predictor of better HRQOL in the diabetes management summary score and the total score. GLM also showed that longer diabetes duration and less than recommended frequency of SMBG were significant predictors of higher diabetes symptom summary score. Income was the only variable to show significant differences in both summary scores and total score in the model. Adolescents coming from families with income higher than 5000 NIS per month reported better scores in both summary scores and the total score compared to those from families with income less than 2000 NIS or 2000–5000 NIS per month. However, significant differences were only present between the lowest and highest category for the summary scores, while for the total score, both adolescents from lower- and middle-income families scored significantly lower than those from higher income ones.

## 4. Discussion

This study aimed to evaluate HRQOL among adolescents with T1D living in the West Bank, Palestine. Among the 170 patients participated in the study, an acceptable mean of 70.6 for the total score was reported. Better scores were reported for the diabetes management summary score compared to the diabetes symptom summary score. Worry and communication were the lowest and highest reported subscores, respectively. Parents reported significantly lower results than adolescents in all scores except for the worry subscore, where they reported significantly higher. Income was the only variable to significantly predict the total score and both summary scores. HbA1c was a significant predictor of both diabetes symptom summary score and total score. Gender differences were significant in the diabetes management summary score and total score. Other factors that had significant relations with some scores were diabetes duration and frequency of glucose monitoring.

Looking at the fact that this study is one of a few to use the new Peds QL 3.2 Diabetes Module, our discussion will mostly include studies using the previous version as no major differences between both tools are present [32]. Reported HRQOL in this study was comparable to that reported in Kuwait and Korea and the global TEENs study [23, 34, 35]. Higher scores were reported in Hungary and Italy [36, 37], while lower scores were reported in India, Greece, Saudi Arabia, and Iran [24, 26, 38, 39].

### 4.1. Factors Associated With HRQOL Among Adolescents With T1D

Several predictors of HRQOL were found in our study. Income was the only factor to predict both summary scores and the total score. Our study showed that adolescents coming from families with a monthly income greater than 5000 NIS (1350 USD) scored better in all categories of the report. Similar findings were reported from India where adolescents from lower socioeconomic backgrounds had poorer HRQOL [38]. Moreover, in other studies where different tools were used, family income alone or as part of the socioeconomic status was also significantly associated with HRQOL among diabetic adolescents [28, 40]. In our context, differences in HRQOL based on income could be attributed to the fact that not all diabetic expenses are covered by insurance, as reported by patients and their families. Although the majority of our patients were insured (87.1%), most of them did not rely solely on their insurance to provide their insulin (62.9%). This is partly because insulin is not regularly readily available at governmental/UN dispensing pharmacies. Moreover, unlike higher-income countries, glucometer devices and test strips are not covered by insurance in Palestine [41]. Hence, the burden of providing insulin and glucose monitoring supplies is often borne by the patient's family. Our findings were confirmed by the global TEENs study, where significantly lower HRQOL was reported by families who needed extra income to pay for medical costs of diabetes [34].

Household income and health insurance coverage were also found to play a role in determining health outcomes of children with other chronic conditions such as asthma, chronic kidney disease, and epilepsy [42]. The association between low income and poorer health outcomes in adolescents can be further explained by its association with poor diets, less physical activity, and lower adoption of healthy behaviors [43, 44].

Furthermore, it is known from global reports that the highest incidence of mortality among children and adolescents with T1D is in low- and lower-middle-income countries [1]. Palestine has been among the lower-middle income countries for many years until the recent upscaling by the World Bank to upper–middle-income country [45]. Hence, the results of this study assures that income is a major predictor of HRQOL among adolescents with T1D in Palestine. Income disparities should be addressed by healthcare systems in Palestine through better insurance coverage and medication availability.

Males scored better than females in all categories of the scale. Significant differences were noted in the total score and the diabetes management summary score. These findings are consistent with several other studies regionally and globally [23, 24, 34, 35]. Adolescent females with T1D in Palestine also scored lower than their male peers in the Peds QL Generic score as reported by Elissa et al. [30]. Despite the fact that the exact reason for these differences is yet to be uncovered, gender differences could be attributed to the hormonal and physical changes females experience at this age as they enter puberty earlier than males [12]. However, in the Arab countries, including Palestine, traditional gender norms and societal misconceptions have also been reported for their debilitating effect on the experiences of adolescent females with diabetes [46]. Not being able to fulfill their traditional role as a wife and a mother and social constrains on leaving the house alone or participating in physical activity are among the misconceptions and cultural norms that force females to keep their diabetes as a secret, consequently affecting their diabetes management and outcomes [42]. Healthcare providers should be made aware of these gender differences, and efforts to minimize the gap by promoting the health of vulnerable adolescent females with T1D should be encouraged [16].

Another important variable that was significantly related to HRQOL is HbA1c level. In our study, higher diabetes symptom summary scores and total scores were both significantly predicted by lower HbA1c results in the GLM. Moreover, similar yet insignificant difference was observed for the diabetes management summary score and most of the subscores. Comparable results were published both regionally and globally by studies using similar and different tools to evaluate HRQOL [23, 26, 28, 34, 47], while others found no difference [24, 35, 39]. In addition, our results support the association reported by Elissa et al. [30] between higher glycemic control and better Peds QL Generic score among adolescents with T1D in Palestine. Unfortunately, a causal relationship between HRQOL and glycemic control cannot be established based on any cross-sectional study including ours. Regardless of causality, the consistency of the correlation between quality of life and glycemic control in the majority of literature confirms the necessity to consider both as equally important factors in diabetes management among adolescents [9, 10, 17, 20]. This recommendation should be earnestly considered in our context where only 20% of adolescents had controlled HbA1c levels of less than 7.5% with a sample mean of 9.3% ± 2.2%. In spite of relying on the last reported HbA1c level by our patients, these uncontrolled results might be partially explained by the infrequent availability of the test in governmental and UN laboratories and the higher costs of performing such tests in private labs as reported by patients and their families.

One of the diabetes management-associated variables was the frequency of SMBG. Based on adolescents' reports, only 35.9% reported checking their glucose levels less than three to four times/day. Interestingly, in our study, adolescents who checked their glucose levels less than recommended scored significantly better than those who tested more frequently in the diabetes symptom summary score. This result was challenging to explain as it is known from the literature that higher frequency of SMBG is associated with better HRQL and glycemic control [34]. In an attempt to explain this opposite outcome, we would have to consider the fact that the frequency of SMBG is not objectively measured and might have been over reported by the majority of the patients. In addition, those who reported better diabetes symptom summary score may have not felt the need to check their blood glucose levels more frequently. Similar attitudes regarding SMBG were found among type 2 diabetes patients in Palestine [41]. Since glucometers and test strips are not covered by insurance and doctors' encounters with each patient are very short due to overloaded clinics, healthcare providers mostly rely on HbA1c levels and urine analysis for making the appropriate modifications on patients' treatment plans rather than educating them on the importance of SMBG [41], all of which affect patients' attitudes toward SMBG.

Longer duration of diabetes was a significant predictor of better HRQOL in the diabetes symptom summary score. This result is in agreement with those published by Al-Akour, Khader, and Shatnawi [28] in Jordan. An opposite outcome was reported by Abdul-Rassoul et al. [23] in Kuwait, where longer diabetes duration was associated with lower HRQOL. This could be attributed to the fact that their study did not solely include adolescents but expanded to include younger children. Other studies did not find any association between duration of diabetes and HRQOL [12, 24]. The vast majority of our participants (86.5%) were using insulin prefilled pens as the main mode for insulin delivery, which explains why the method of insulin delivery was not with a predictor of HRQOL in our study. Despite the fact that using an insulin pump is widely known to have benefits on diabetes management in adolescents [48], in Palestine, insulin pumps are not covered by insurance and are very expensive to buy and maintain. Only 11 of the 170 interviewed adolescents had insulin pumps, and the majority of them were covered by donations.

### 4.2. Adolescents Versus Parent Reports

In accordance with the present body of literature, our study showed significant disagreement between adolescents and parents reports [12, 23, 24, 26]. Parents consistently underreported their adolescents' HRQOL in all categories of the scale except for the worry subscore. Cognitive development and maturity can explain differences between parents and adolescents' emotions and behaviors [49]. These discrepancies highlight the importance of not relying only on parents when inquiring about adolescents' health. In addition, healthcare providers should be aware of these differences since parents' perceptions have an undeniable effect on adolescents' utilization of healthcare services and diabetes management at large [21, 22].

### 4.3. Strength and Limitations

This study is the first to evaluate HRQOL among adolescents with T1D in Palestine using a validated tool specifically designed to inquire about diabetes-related features. Moreover, our study is the first to include both adolescents and parents' perspectives. Additionally, our sample size of 170 adolescents and their parents was acceptable in light of the lack of national numbers or registries of T1D adolescents and the difficulties faced in trying to locate them. Additionally, we had an impressive participation rate of 89.5%.

There are some limitations that could have affected the results of this research. First, our sample only included adolescents from the northern West Bank, and those attending healthcare centers (HCCs), this must be considered when trying to generalize the results of this study to all adolescents with T1D in Palestine. However, given the fact that in Palestine there is no available registry for T1D patients and the relatively small area of the West Bank which gives expectedly minimal differences between governorates, sample was satisfying. Second, although our results show acceptable HRQOL with regard to other countries in the region and abroad, this might be due to the use of a standardized tool that quantitatively describes HRQL. However, the Palestinian context is more complex than many of the countries that have used Peds QL tools. Prolonged military occupation and several related restrictions on movement of people and goods, access to healthcare services and education, and disruptions of daily lives and family relations are all considered barriers that affect the quality of life of all Palestinians [50]. Hence, a more in-depth inquiry about the effects of these political challenges should be further explored through future qualitative research, which will enable a deeper understanding of the experience of T1D adolescents under these conditions. Other factors that were not considered in this study are the mental health factors and family dynamics impact on HRQOL. Third, many of the diabetes-related factors such as HbA1c, frequency of SMBG, and frequency of clinic visits were not objectively measured rather reported by the patients themselves; hence, the results of these relations are subject to recall bias. Finally, the cross-sectional design of our study does not allow for assuming causality between any of the variables that predicted HRQOL. Future longitudinal studies are encouraged for these purposes.

## 5. Conclusion

Adolescents with T1D in Palestine had an acceptable level of HRQOL using the Peds QL 3.2 Diabetes Module. Parents reported significantly lower HRQOL than adolescents. Male adolescents, those who had lower HbA1c levels, and came from high-income families reported significantly better HRQOL than their peers. We recommend that healthcare providers consider health-promoting activities that are specifically designed to improve HRQOL among vulnerable groups of females and those from poorer backgrounds. Future national health strategies should consider income differences and try to overcome health gaps among adolescents with T1D coming from low-income families through better insurance plans and specific measure related to access and follow-up. Future research is needed to explore the political and cultural aspects and their effects on HRQOL among diabetic adolescents in Palestine.

## Figures and Tables

**Table 1:** General characteristics of the study participants (*n* = 170).

Variables	Parameters	M (SD)	*n*	%
Demographic variables				
Age	—	13.58 (2.30)	—	—
Sex	Female Male	—	100 70	58.8 41.2
Place of residence	Rural Urban Camp	—	54 103 13	31.8 60.6 7.6
Diabetes specific variables				
Diabetes duration (years)	—	4.76 (3.21)	—	—
Method of insulin delivery	Pen Pump Syringes	—	147 11 12	86.5 6.5 7.1
HbA1c	<7.5 ≥7.5	—	34 135	20.0 79.9
Frequency of SMBG	<3–4 times/day ≥3–4 times/day	—	61 109	35.9 64.1
Health care variables				
Medical insurance	Insured Not insured Multiple insurance	—	148 7 15	87.1 4.1 8.8
Type of diabetes clinic	Governmental Private UN More than one provider	—	76 44 1 49	44.7 25.9 0.6 28.8
Frequency of visits to diabetes clinic	<3 months Every 3 months Every 4–6 months >6 months	—	63 79 16 12	37.1 46.5 9.4 7.1
Diabetes medication provider	Governmental/UN Private Donations More than one provider	—	31 29 3 107	18.2 17.1 1.8 62.9
Family variables				
Monthly income (NIS)	<2000 2000–5000 >5000	—	31 114 25	18.2 67.1 14.7
Number of family members	1–2 3–4 5–6 >6	—	1 13 75 81	0.6 7.6 44.1 47.6

Abbreviations: HbA1c, hemoglobin A1c; SMBG, self-monitoring of blood glucose.

**Table 2:** HRQOL scores for adolescent patient and parent reports.

Peds QL scores	Patient report		Parent report		Difference	Wilcoxon signed-rank test		
	Mean	SD	Mean	SD		*z*	95%CI (lower, upper)	*p* value
Diabetes symptoms	64.37	15.01	64.50	16.12	0.13	−0.90	63.44, 65.30	0.931
Diabetes management	75.90	14.62	71.36	17.41	4.54	−3.55	75.90, 75.90	<0.001
Treatment barriers	72.588	18.74	61.735	24.03	10.85	−5.37	72.58, 72.58	<0.001
Treatment adherence	81.22	17.73	78.08	17.89	3.14	−2.46	81.20, 81.23	0.014
Worry	62.01	24.64	71.22	27.10	9.21	−4.09	62.01, 62.01	<0.001
Communication	82.94	22.61	73.71	31.27	9.23	−3.83	82.94, 82.94	<0.001
Total score	70.61	12.77	68.22	14.60	2.39	−2.56	70.60, 70.62	0.009

Abbreviations: HRQOL, health-related quality of life; SD, standard deviation.

**Table 3:** Predictors of adolescents' HRQOL (generalized linear model).

Parameter	Predictor	Diabetes symptom summary score*⁣*^*∗*^				Diabetes management summary score*⁣*^*∗∗*^				Total score*⁣*^*∗∗∗*^			
		B	SE	95% Wald CI (lower, upper)	*p* value	B	SE	95% Wald CI (lower, upper)	*p* value	B	SE	95% Wald CI (lower, upper)	*p* value
Intercept	—	4.64	0.15	4.35, 4.93	<0.001	4.57	0.12	4.34, 4.81	<0.001	4.59	0.11	4.37, 4.81	<0.001
Gender	Female vs. male	−0.05	0.04	−0.12, 0.03	0.216	−0.06	0.03	−0.13, −0.00	0.039	−0.06	0.03	−0.12, −0.00	0.046
Age	—	−0.02	0.01	−0.04, 0.00	0.054	−0.01	0.01	−0.02, 0.01	0.504	−0.01	0.01	−0.02, 0000	0.146
Income	<2000 vs. >5000	−0.15	0.07	−0.29, −0.02	0.024	−0.12	0.05	−0.23, −0.01	0.036	−0.13	0.05	−0.23, −0.03	0.011
	2000–5000 vs. >5000	−0.09	0.05	−0.21, 0.01	0.069	−0.09	0.04	−0.17, 0.00	0.055	−0.09	0.04	−0.17, −0.01	0.028
Duration of diabetes	—	0.01	0.01	0.00, 0.02	0.041	0.01	0.00	−0.00, 0.01	0.214	0.01	0.00	0.00, 0.02	0.057
HbA1c	—	−0.02	0.01	−0.04, −0.01	0.007	−0.01	0.01	−0.02, 0.00	0.142	−0.01	0.01	−0.03, −0.00	0.018
Frequency of SMBG	<3−4 times/day vs. ≥3−4 times/day	0.10	0.04	0.02, 0.20	0.021	0.01	0.36	−0.06, 0.08	0.776	0.05	0.03	−0.02, 0.01	0.149

Abbreviations: HbA1c, hemoglobin A1c; SMBG, self-monitoring of blood glucose.

*⁣* ^*∗*^Model summary: (a) goodness of fit (deviance/df = 0.062, Pearson chi-square/df = 0.052). (b) Omnibus test (χ^2^(6) = 19.809, *p*=0.006).

*⁣* ^*∗∗*^Model summary: (a) goodness of fit (deviance/df = 0.041, Pearson chi-square/df = 0.035). (b) Omnibus test (χ^2^(6) = 12.870, *p*=0.075).

*⁣* ^*∗∗∗*^Model summary: (a) goodness of fit (deviance/df = 0.036, Pearson chi-square/df = 0.030. (b) Omnibus test (χ^2^(6) = 19.776, *p*=0.006).

## Data Availability

The datasets used in this study are available from the corresponding author upon reasonable request.
